# Selective targeting of HDAC1/2 elicits anticancer effects through Gli1 acetylation in preclinical models of SHH Medulloblastoma

**DOI:** 10.1038/srep44079

**Published:** 2017-03-09

**Authors:** Sonia Coni, Anna Barbara Mancuso, Laura Di Magno, Giulia Sdruscia, Simona Manni, Silvia Maria Serrao, Dante Rotili, Eleonora Spiombi, Francesca Bufalieri, Marialaura Petroni, Monika Kusio-Kobialka, Enrico De Smaele, Elisabetta Ferretti, Carlo Capalbo, Antonello Mai, Pawel Niewiadomski, Isabella Screpanti, Lucia Di Marcotullio, Gianluca Canettieri

**Affiliations:** 1 Department of Molecular Medicine, SAPIENZA Unversity of Rome, Viale Regina Elena 291, 00161, Rome, Italy; 2 Center for Life Nano Science@Sapienza, Istituto Italiano di Tecnologia, Viale Regina Elena 291, 00161, Roma, Italy; 3 Department of Drug chemistry and Technologies, SAPIENZA University of Rome, P.le A. Moro 5, 00185, Rome, Italy; 4 Department of Cell Biology, Nencki Institute of Experimental Biology, 02-093, Warszawa, Poland; 5 Department of Experimental Medicine, SAPIENZA, University of Rome, Viale Regina Elena 324, 00161, Rome, Italy; 6 Pasteur Lab, Department of Molecular Medicine, Sapienza University of Rome, Viale Regina Elena 291, Italy; 7 Neuromed Institute, Pozzilli, 86077, Italy

## Abstract

SHH Medulloblastoma (SHH-MB) is a pediatric brain tumor characterized by an inappropriate activation of the developmental Hedgehog (Hh) signaling. SHH-MB patients treated with the FDA-approved vismodegib, an Hh inhibitor that targets the transmembrane activator Smoothened (Smo), have shown the rapid development of drug resistance and tumor relapse due to novel Smo mutations. Moreover, a subset of patients did not respond to vismodegib because mutations were localized downstream of Smo. Thus, targeting downstream Hh components is now considered a preferable approach. We show here that selective inhibition of the downstream Hh effectors HDAC1 and HDAC2 robustly counteracts SHH-MB growth in mouse models. These two deacetylases are upregulated in tumor and their knockdown inhibits Hh signaling and decreases tumor growth. We demonstrate that mocetinostat (MGCD0103), a selective HDAC1/HDAC2 inhibitor, is a potent Hh inhibitor and that its effect is linked to Gli1 acetylation at K518. Of note, we demonstrate that administration of mocetinostat to mouse models of SHH-MB drastically reduces tumor growth, by reducing proliferation and increasing apoptosis of tumor cells and prolongs mouse survival rate. Collectively, these data demonstrate the preclinical efficacy of targeting the downstream HDAC1/2-Gli1 acetylation in the treatment of SHH-MB.

Medulloblastoma (MB) is the most frequent brain malignancy of the childhood, with an incidence rate in children of approximately 6 per million^1^.

Despite the current radical treatment, which combines surgery, radiation and chemotherapy, MB is still associated to 30% of lethality. Moreover, survivors usually develop severe neurological side effects, such as ataxia and cognitive deficits, underscoring the importance to find alternative therapeutic strategies^2^.

Whole genome sequencing approaches have led to the identification of 4 different molecular subgroups of MB, based on the genetic lesions/altered pathway found: WNT, SHH, Group C and Group D^3^. The identification of specific molecular alterations has opened the door to personalized, pathway-targeting strategies, leading to the first clinical achievement, obtained with the SHH subgroup (SHH-MB).

In this group, which accounts for about 30% of total MBs, tumors are characterized by the inappropriate expression of genes that are transcriptionally regulated by the developmental Hedgehog (Hh) signaling^4^. In normal cells, this pathway is activated upon interaction of the Shh ligand with the inhibitory Patched (Ptch1) receptor. This leads to the de-repression of the transmembrane transducer Smoothened (Smo), which is followed by a sequence of events that involves the cytoplasmic inhibitor Suppressor of Fused (SuFu) and terminates with the activation of Gli transcription factors (Gli1, Gli2, Gli3)^5^.

Genetic alterations found in the SHH-MB subgroup, include mutations of *Ptch1, Smo* or *Sufu* or amplifications of *Shh* or *Gli2* genes^6^. In all cases, the overall consequence of these alterations is the hyperactivation of the pathway, which represents a crucial step for this type of malignancy. This notion has led to the discovery of the inhibitor vismodegib, the first anti-Hedgehog drug approved by the FDA for the treatment of metastatic or recurrent locally advanced Basal Cell Carcinoma (BCC)^7^ and, currently, in clinical trials for SHH-MB. In two separate phase II clinical trials, patients with recurrent or refractory SHH-MB or non-SHH-MB have been treated with vismodegib^8^. In a subset of SHH-MB patients, vismodegib displayed a short-term clinical efficacy, increasing progression free survival. However, all patients eventually developed drug resistance, likely linked to novel mutations or activation of compensatory pathways that restore downstream activation. Moreover, SHH-MB patients with mutations of genes downstream of Smo did not show any benefit with vismodegib treatment. Therefore, these results clearly indicate that alternative approaches, preferably targeting downstream factors are better options to treat MB.

Compounds with ability to direct bind and inhibit Gli activity, such as GANT61^9^, ATO^10^,^11^ and GlaB^12^ have shown efficacy against SHH-MB growth in preclinical models. However, toxicity and specificity are still being investigated for these drugs and further pharmacological studies are still required before they can enter clinical trials^13^. Alternatively, indirect inhibitors, mostly affecting Gli post-translational modifications, or inhibitors of key pathways regulated by Hh/Gli, could be used for the same purpose^5^,^14^.

In previous studies, we have observed that Gli1 and Gli2 are acetylated proteins, being this modification a key regulatory checkpoint, regulating Hh transcriptional output^15^,^16^.

Acetylation of Gli1 and Gli2 inhibits their transcriptional activity by preventing the recruitment of the two transcription factors to target promoters^16^, thus representing an attractive druggable target.

Gli acetylation is catalyzed by the histone acetyl-transferase p300 and is removed by HDAC1 and HDAC2. Notably both HDAC1 and HDAC2 are induced by Hh signaling, engaging a positive loop, and are consequently found upregulated in SHH-MB^15^,^17^. Therefore, these observations suggest that targeting the two HDACs and promoting Gli acetylation could be a successful approach to counteract SHH-MB growth.

We report here the effect of the selective genetic and pharmacological inhibition of HDAC1 and HDAC2 *in vitro* and in preclinical models of SHH-MB growth. Importantly, we illustrate the specificity of this mechanism in targeting Gli1 acetylation, thereby providing the first demonstration of the relevance of this approach for the treatment of SHH-MB.

## Results

### Ablation of HDAC1 and HDAC2 inhibits Hh signaling and decreases SHH-MB cell proliferation

**Figure 1:**
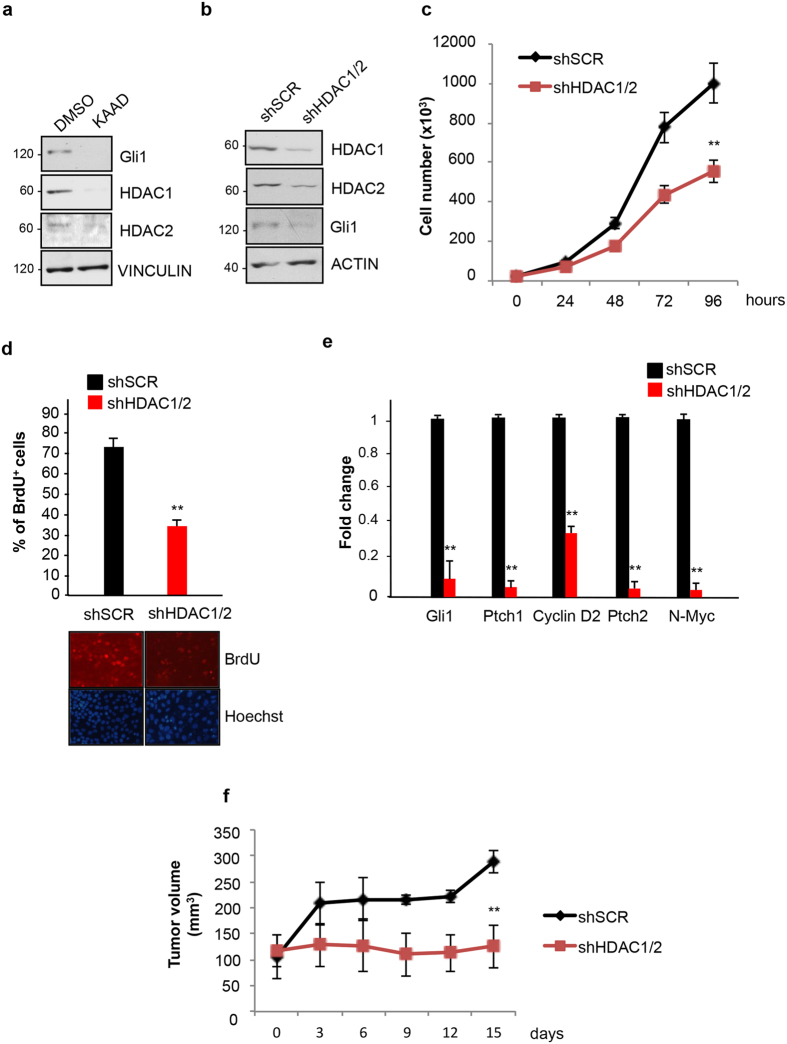
Selective HDAC1/2 knockdown counteracts Hedgehog-dependent medulloblastoma growth *in vitro* and *in vivo*. (**a**) Gli1, HDAC1, HDAC2 protein levels in Med1-MB cells treated for 72 h with 1 μM Kaad-cyclopamine or DMSO as a control. Vinculin, loading control. (**b**) Western blot showing HDAC1/2, Gli1, actin levels from Med1-MB cells transduced with lentiviruses expressing shRNAs for HDAC1 and HDAC2 (shHDAC1/2) or scrambled (shSCR). (**c**) Growth curve in Med1-MB cells after stable lentiviral mediated knock down of HDAC1 and HDAC2. n = 3 (**d**) BrdU incorporation assay in HDAC1/2-deficient Med1-MB cells (see above). Bottom, representative pictures of BrdU/Hoechst staining (**e**) QPCR analysis of Gli1, Ptch1, Cyclin D2, Ptch2, N-Myc mRNAs in HDAC1/2-deficient Med1-MB cells. The experiments were performed in triplicate. n = 4 (**f**) Stable HDAC1/2-deficient Med1-MB (shHDAC1/2) cells or control cells expressing a non-specific scrambled shRNA (shSCR) were grafted subcutaneously into mice flanks (2 × 10^6^ /flank). Tumor volumes were measured with calipers as indicated. (n = 6 for each experimental group). *p < 0.05; **p < 0.01. Uncropped Western blot gels related to this figure are displayed in Suppl. Fig. 4.

Previous studies demonstrated that the levels of HDAC1 and HDAC2 are elevated in SHH-MB. Since Hh activation induces an increase of HDAC1/2 protein levels, we first tested whether the observed HDACs overexpression is linked to the aberrant Hh signaling that typically characterizes SHH-MB subgroup. To this end, we used the Med1-MB cell line generated from a spontaneous tumor arisen in a *Ptch1*^+/−^; *lacZ mouse*^18^,^19^. This mouse model carries a heterozygous deletion of *Ptch1* gene, which is often found mutated in sporadic and familiar SHH-MB. Since Ptch1 is a repressor of the Hh pathway, its monoallelic deficiency causes a constitutive activation of the signaling and the occurrence of Medulloblastoma in about 15% of the carriers^20^. Indeed, Med1-MB cells display an active Hh signaling that can be turned off by selective Hh inhibitors^18^. Confirming this notion, exposure of Med1-MB cells to the Smo antagonist Kaad-cyclopamine^21^ caused a marked decrease of Gli1, a standard Hh target. This inhibition was accompanied to a parallel reduction of HDAC1 and HDAC2 protein levels, confirming that these deacetylases are functionally connected to the Hh signaling (Fig. 1a). To determine whether this functional connection is required for the proliferation of SHH-MB cells, we performed a stable lentiviral-mediated knockdown of the two HDACs. As shown in Fig. 1b,c,d, ablation of both HDACs caused a marked reduction of the proliferation rate and incorporation of BrdU compared to cells infected with a virus expressing a non-specific, scrambled shRNA. The decreased proliferation was also associated to a marked suppression of the Hh targets *Gli1, Ptch1, CyclinD2, Ptch2 and N-Myc* (Fig. 1e), demonstrating the ability of the two HDACs to promote Hh-mediated proliferation and gene expression *in vitro*.

Similar results were obtained in primary cultures of SHH-MB, derived from Math1-Cre/Ptc^fl/fl^ mice, which lack both *Ptch1* alleles in Math1-expressing Granule Cell Progenitors (GCPs) and develop SHH-MB by the 10th week of age^22^. With these cells, we observed a decrease of HDAC1/2 levels upon exposure to Kaad-cyclopamine and a significant decrease of cell proliferation after HDAC1/2 knockdown (Supplementary Fig. S1).

To evaluate the effect of HDACs ablation on tumor growth *in vivo*, we grafted the stable HDAC1/2-deficient and control Med1-MB cells into flanks of athymic nude CD1 mice and monitored the growth of the tumor volume over time. SHH-MB cells lacking HDACs grew slower and at the end of the experiment were significantly smaller than controls, demonstrating the requirement of the two deacetylases for Hh-dependent tumor growth (Fig. 1f).

Collectively, these data demonstrate that HDAC1/2 are regulated by the Hh signaling to support tumor cell proliferation and argue that a selective pharmacological targeting of these deacetylases may counteract SHH-MB growth.

### Pharmacological blockade of HDAC1/2 inhibits Hh signaling, cell proliferation and promotes apoptosis in SHH-MB cells

**Figure 2:**
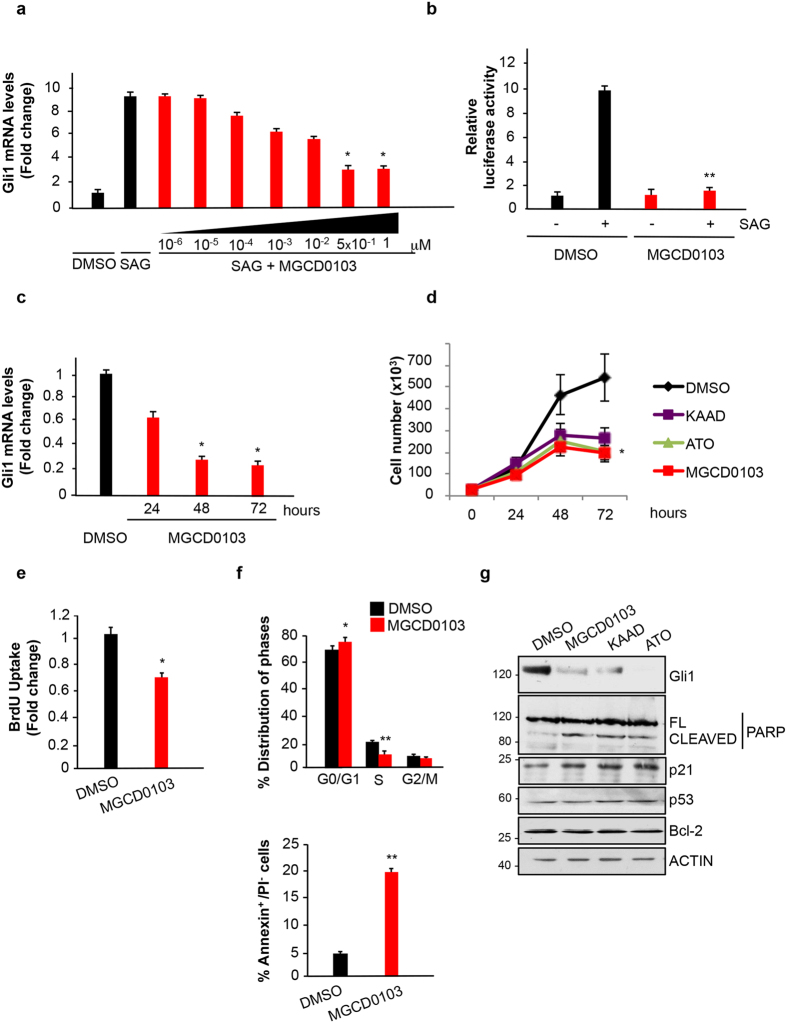
HDAC1 and HDAC2 pharmacological inhibition suppresses Hedgehog function. (**a**) MEF cells were treated with SAG and increasing concentrations of MGCD0103 for 24 hours as indicated and Gli1 mRNA levels were analyzed by QPCR. DMSO, untreated control. Experiments were performed in triplicates. n = 3 (**b**) Luciferase assay in Shh-Light II cells treated with SAG and/or MGCD0103 for 24 hours. DMSO, untreated control. The experiments were performed in triplicates. n = 3 (**c**) MEF Ptch1^−/−^ were treated with MGCD0103 or DMSO as a control for 24, 48 or 72 hours. Gli1 mRNA levels were analyzed by QPCR, experiments were performed in triplicates. n = 3 (**d**) Growth curve of Med1-MB cells treated with 1M Kaad-cyclopamine, 5 μM ATO, 0.5 μM MGCD0103 or DMSO for the indicated times. Each experimental point was performed in triplicate. n = 4 (**e**) BrdU incorporation assay in Med1-MB cells treated for 24 hours with 0.5 μM MGCD0103 or DMSO. (**f**) Cell cycle profile of Med1-MB cells treated for 24 h with 0.5 μM MGCD0103 or DMSO (Top). Flow cytometry analysis of Med1-MB cells double stained with annexin V/propidium iodide after treatment with 0.5 μM MGCD0103 or DMSO for 48 h (bottom). n = 3 (**g**) Western blot analysis in Med1-MB cells after 48 hours of treatment with 1 μM Kaad-cyclopamine, 0.5 μM MGCD0103, 5 μM ATO or DMSO as a control. Protein levels of Gli1, cleaved/full length (FL) PARP, p21, p53, Bcl-2 and actin are shown. *p < 0.05; **p < 0.01. Uncropped Western blot gels related to this figure are displayed in Suppl. Fig. 5.

Mocetinostat (MGCD0103) is an isotype inhibitor of class I HDACs, selective for HDAC1/2 within the nanomolar concentrations, which inhibits the growth of different tumors in preclinical and clinical settings^23^,^24^. Thus, we studied whether this drug elicits the same inhibitory effect on Hh-dependent function observed upon HDAC1/2 depletion. We first tested its effect on Hh signaling in MEF cells treated with the Smo agonist SAG^25^, monitoring the levels of the Hh target gene Gli1 by real time QPCR. Exposure of cells to SAG induced a marked elevation of Gli1 mRNA levels that was significantly reduced upon exposure to MGCD0103 (Fig. 2a). The maximal inhibition was at 0.5 μM, corresponding to the reported concentration that selectively inhibits HDAC1 and HDAC2^26^. Thus, we used this concentration for all the subsequent experiments.

Inhibition of the Hh signaling was observed also in the Shh-Light II cells, a mouse fibroblast stable cell line harboring 8xGli luciferase and renilla reporters^21^, where MGCD0103 reduced the SAG-induced luciferase activity (Fig. 2b). Similarly, in Ptch1^−/−^ MEF cells, where the Hh signaling is constitutively active and the Hh target genes are consequently upregulated^20^, MGCD0103 caused a marked, time-dependent decrease of Gli1 mRNA levels (Fig. 2c).

We then studied whether MGCD0103 inhibits the *in vitro* growth of SHH-MB cells. Treatment of Med1-MB cells with this drug led to a marked inhibition of the growth rate that was comparable to the inhibition achieved with the Smo inhibitor Kaad cyclopamine or with the Gli inhibitor arsenic trioxide (ATO)^10^,^11^ (Fig. 2d).

To determine whether the inhibitory effect of MGCD0103 is linked to inhibition of cell proliferation, we performed BrdU staining. As shown in Fig. 2e, MGCD0103 caused a significant decrease of proliferating, BrdU-positive cells compared to control. Analysis of cell cycle distribution by flow cytometry revealed that MGCD0103 induces a significant depletion of cells in S phase, accompanied by an increase of cells in G1 phase (Fig. 2f, upper panel).

To study if MGCD0103 also promotes apoptosis, we performed Annexin V-Propidium Iodide (PI) double staining. As shown in Fig. 2f (bottom panel), MGCD0103 induced an increase in the percentage of Annexin V-positive cells in the PI-negative population.

These results indicate that MGCD0103 exerts a dual effect, by inhibiting cell proliferation and promoting apoptotic cell death in Med1-MB cells.

We next analyzed key regulators of cellular proliferation and apoptosis that were found modulated by HDAC inhibitors in other tumors: p21, p53, Bcl-2 and PARP^27^. Cleaved PARP and p21 were induced by MGCD0103 (Fig. 2g), supporting the flow cytometry data of G1 accumulation, S-phase depletion and increased apoptosis. The same increase of cleaved PARP and p21 was also observed with the Hh inhibitors KAAD or ATO, whereas neither MGCD0103 nor Hh inhibitors affected p53 and Bcl-2 levels (Fig. 2g), indicating that these compounds limit tumor growth by modulating common targets.

Collectively, the above results demonstrate that MGCD0103 inhibits Hh-dependent transcriptional response and limits SHH MB cell growth, by affecting both cell proliferation and apoptosis.

### The effect of Mocetinostat on Hh-regulated functions is dependent on Gli1 acetylation

Having observed that MGCD0103 restrains the growth of SHH-MB cells, where a key tumorigenic driver is upregulated Gli1^28^, we wondered whether the inhibitory effect was linked to the acetylation of Lysine 518, a modification that causes inhibition of Gli1 transcriptional activity, by preventing its promoter occupancy^16^. Indeed, HDAC1/2 deacetylate and enhance Gli1 transcriptional activity, thus suggesting that, by blocking these enzymes, MGCD0103 may inhibit Hh function via Gli1 acetylation. Since in a recent work it was also observed that HDAC6 inhibits Hh signaling by acting both upstream and downstream of Sufu^29^, we first tested at what level the inhibitory effect of MGCD0103 occurs. To this end, we used the Sufu^−/−^ MEF cells, where the absence of the cytoplasmic inhibitor Sufu causes an increase of Hh target gene expression, independently of the receptor (Ptch1/Smo) activation^30^.

**Figure 3:**
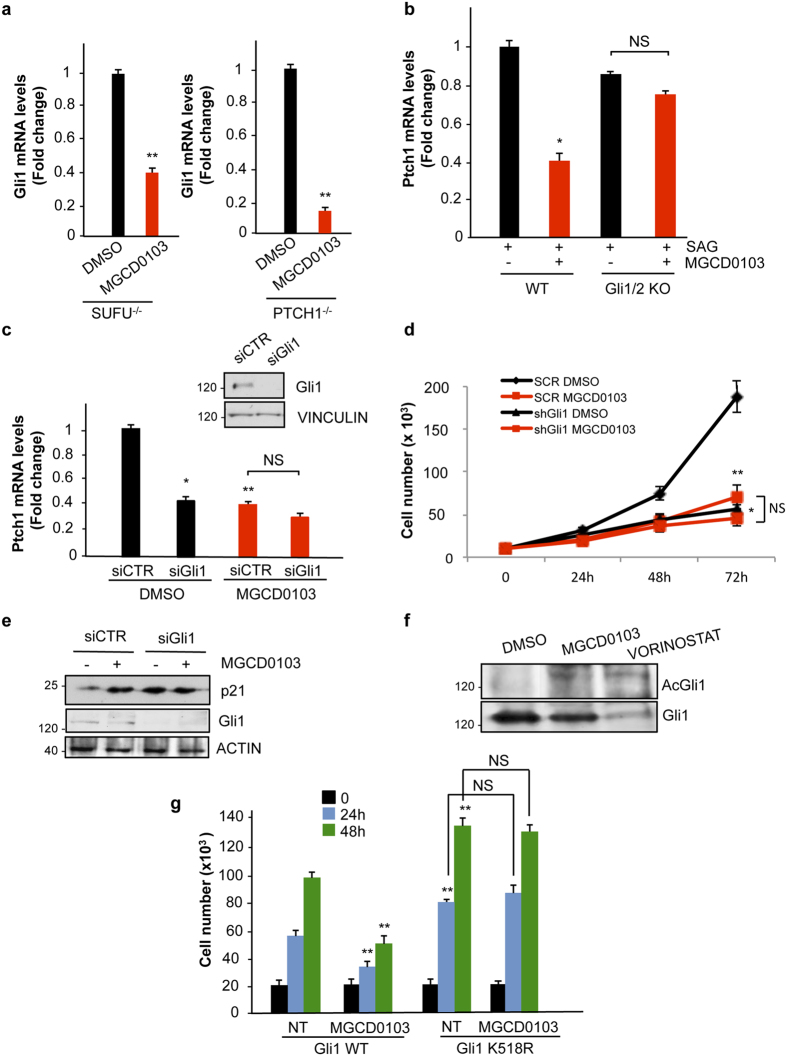
The effect of HDAC1 and HDAC2 inhibition depends on Gli K518 acetylation. (**a**) QPCR analysis of Gli1 mRNA levels in MEF Sufu^−/−^ and MEF Ptch1^−/−^ treated with 0.5 μM MGCD0103 for 24 hours. Each experiment was performed in triplicate. n = 3 (**b**) WT or Gli1 and Gli2 KO MEF cells were treated with SAG for 48 h and/or 0.5 μM MGCD0103 for the last 16 h and QPCR analysis of Ptch1 mRNA levels was performed in triplicate. n = 3 (**c**) QPCR analysis of Ptch1 mRNA in Med1-MB cells expressing siRNA for Gli1 (siGli1) or si control RNA (siCTR) for 96 h and then treated with 0.5 μM MGCD0103 for 24 h. Upper panel, western blot analysis indicating the efficacy of Gli1 knockdown. Vinculin was used as loading control. n = 4 (**d**) Growth curve of Med1-MB cells expressing siRNAs targeting Gli1 (siGli1) or control siRNA (siCTR) for 96 h and then treated with 0.5 μM MGCD0103 or DMSO for the indicated time. Each experimental point was performed in triplicate. n = 3 (**e**) Western blot analysis of p21 in Med1-MB cells expressing siGli1 or siCTR RNA for 96 h and treated with 0.5 μM MGCD0103 or DMSO as a control for 48 h. Gli1 levels are shown as control of knockdown efficacy. (**f**) Acetylation of endogenous Gli1 in Med1-MB treated with 0.5 μM MGCD0103 or 100 nM vorinostat or DMSO. Total Gli1 levels are shown (**g**) Growth curve in DAOY cells stably transfected with Gli1 WT or Gli1 K518R mutant. Cells were seeded in triplicates, treated with MGCD0103 or DMSO as control and counted at the indicated time points. n = 3 *p < 0.05; **p < 0.01. Uncropped Western blot gels related to this figure are displayed in Suppl. Fig. 6.

As shown in Fig. 3a, MGCD0103 strongly inhibited Gli1 mRNA expression in Sufu^−/−^ cells and the effect was comparable to that obtained in Ptch1^−/−^ MEF cells. This indicated that MGCD0103 entirely acts downstream of Sufu, likely by affecting Gli function.

To confirm that the effect is dependent on Gli, we used Gli1/Gli2 KO MEF cells. These cells lack both Gli1 and Gli2 alleles (Supplementary Fig. S2) but retain some Hh inducibility due to the presence of Gli3^31^. Since Gli1 and Gli2, but not Gli3, have a conserved acetylatable lysine^15^, we expected that these cells were not sensitive to MGCD0103 treatment. In keeping with our hypothesis, MGCD0103 failed to inhibit Hh-mediated transcription of *Ptch1* mRNA in Gli1/2 KO MEF cells (Fig. 3b), whereas it significantly reduced its expression in the WT counterpart. Similarly, in Med1-MB cells, where Gli1 is overexpressed, while Gli2 levels are barely detectable (Supplementary Fig. S2), siRNA-mediated knockdown of Gli1 (siGli1) abrogated MGCD0103 inhibition of the Hh target gene *Ptch1*, compared to cells transfected with the non-specific siRNA (siCTR) (Fig. 3c). In keeping with these data, MGCD0103 treatment did not induce any further inhibition of tumor growth or upregulation of p21 in Gli1-deficient cells (Fig. 3d,e), indicating that the antitumor properties of this drug are dependent on Gli1 in these cells.

To determine whether MGCD0103 induces Gli1 acetylation, we performed western blot analysis with an antisera specifically reacting against the acetylated K518 of Gli1^16^. As shown in Fig. 3f we observed that MGCD0103 and the Pan HDAC inhibitor vorinostat increased endogenous Gli1 acetylation, thus suggesting that this modification may account for the observed inhibitory effect.

To address this issue, we generated a MB cell line stably expressing WT Gli1 or its non-acetylatable K518R mutant (Fig. S2). Exposure to MGCD0103 significantly inhibited the proliferation rate of cells expressing WT Gli1. In contrast, in cells expressing Gli1 K518R, the proliferation rate was higher than those expressing WT Gli1 but was no longer inhibited by MGCD0103 (Fig. 3g). This latter evidence demonstrates that MGCD0103 inhibits Gli1-driven MB cell proliferation via K518 acetylation.

### MGCD0103 displays strong anticancer effect on mouse models of SHH-MB

**Figure 4:**
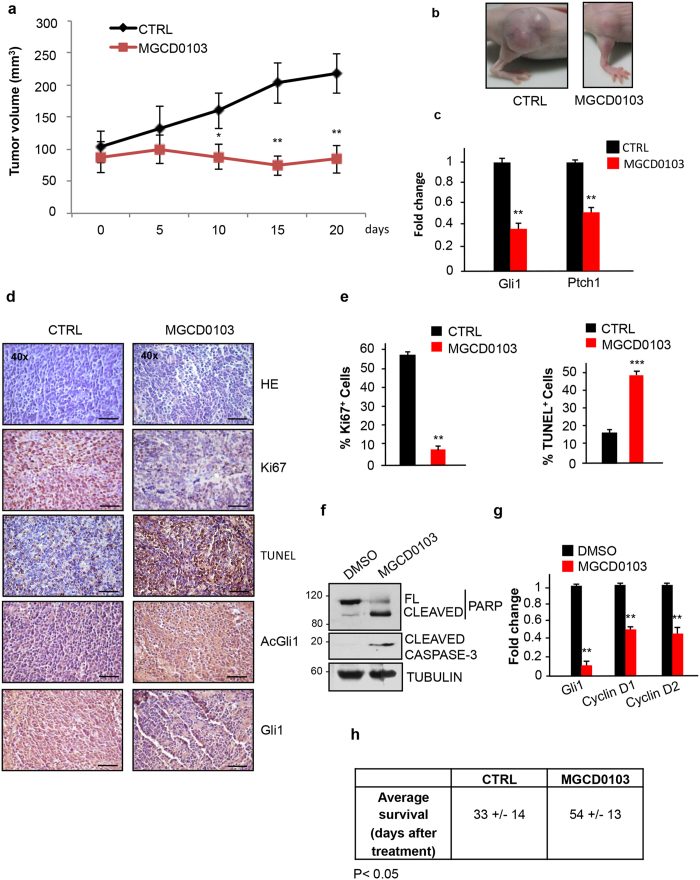
Pharmacological inhibition of HDAC1 and HDAC2 counteracts Shh-dependent medulloblastoma growth *in vivo*. (**a**) Primary SHH-MB cells were grafted into nude mice (2 × 10^6^ cells/flank). After two weeks from the injection, when the tumor volumes reached 100 mm^3^, mice were treated daily with 170 mg/Kg MGCD0103 or H_2_O *per os.* The tumor masses were measured with a caliper at the indicated times. (n = 8 for each experimental group) (**b**) Representative pictures of tumor masses at the end of the experiment (CTR vs MGCD0103) (**c**) QPCR analysis of Gli1 and Ptch1 mRNA levels from pooled RNA samples extracted from the explanted tumor masses of each experimental group. Results were performed in triplicates. (**d**) Immunohistochemical staining of Gli1, AcGli1, Ki67, and Tunel assay in sections from the allografted tumors. Hematoxylin/Eosin staining was performed to control the quality of the slides (scale bar, 100 μm). (**e**) Analysis of the percentage of Ki67 positive cells and percentage of apoptotic cells from the sections in Fig. 4d. (**f**) Western blot analysis on MB samples (pools of 4 samples for each experimental group) excised from Math1-Cre/Ptc^fl/fl^ mice subcutaneously treated for 6 hours with DMSO or MGCD0103 (170 mg/Kg). Apoptosis was evaluated by analyzing PARP and caspase 3 processing. Tubulin, loading control. (**g**) QPCR analysis of Gli1, Cyclin D1 and Cyclin D2 levels of MB samples from Fig. 4f (**h**) Average survival of Math1-Cre/Ptc^fl/fl^ mice treated with MGCD0103 (80 mg/kg) or H_2_O *per os* starting from 4 weeks of age. MGCD0103 (n = 6) CTRL (n = 6). ***p < 0.001; **p < 0.01; *p < 0.05. Uncropped Western blot gels related to this figure are displayed in Suppl. Fig. 7.

We tested the ability of MGCD0103 to inhibit the growth of SHH-MB in *in vivo* models. Tumors spontaneously generated in Math1-Cre/Ptc^fl/fl^ mice were explanted, disaggregated and subcutaneously grafted into flanks of athymic nude mice. When the tumor reached 100 mm^3^, 170 mg/Kg MGCD0103 was orally administered to mice every day and tumor volumes were measured over time. As shown in Fig. 4a,b and S3, MBs grew slower and were significantly smaller at the end of the experiment in MGCD0103-treated mice compared to control littermates. mRNA levels of the Hh target genes *Gli1* and *Ptch1* were markedly reduced in tumor cells from MGCD0103-treated mice (Fig. 4c), documenting the inhibition of Hh signaling. This down-regulation was associated to a significant 4 fold decrease of cell proliferation and 2.5 fold increase of apoptosis in MGCD0103-treated mice as shown by the Ki67 and Tunel assays, respectively, of tumor sections (Fig. 4d,e). Supporting the role of K518 acetylation in this process, the staining with acetylated Gli1 antisera was greatly increased, while the levels of total Gli1 were reduced in MGCD0103-treated mice, as a consequence of the pathway inhibition (Fig. 4d).

To determine whether MGCD0103 is also effective on spontaneously originated, intracranial SHH-MB, we injected Math1-Cre/Ptc^fl/fl^ mice showing clinical signs of MB (i.e. ataxia, lack of coordination, reduced motility) with a bolus of MGCD0103 or vehicle for 6 hours. Real time QPCR and immunoblots analyses demonstrated that MGCD0103 crosses the blood-brain barrier, represses Hh-mediated transcription and promotes apoptosis in tumor cells (Fig. 4f,g). By contrast, while the acetylated levels of histone H3 (AcH3) were increased, witnessing the efficacy of the MGCD0103 treatment, no PARP processing was detected in normal cerebella of WT littermates, indicating the tumor-specificity of this effect (Supplementary Fig. S3).

To study if MGCD0103 prolongs the survival of SHH-MB-prone mice, we treated Math1-Cre/Ptc^fl/fl^ with orally administered MGCD0103, starting from 4 weeks of age. As shown in Fig. 4h, the average survival was significantly increased, by 60%, in MGCD0103-treated mice compared to controls, demonstrating the therapeutic benefit of this drug in tumors developed *in situ*.

Collectively, these data demonstrate the efficacy of MGCD0103 in restraining SHH-MB growth by inducing an acetylation-dependent Hh inhibition in preclinical models.

## Discussion

In the present work we have demonstrated the vulnerability of SHH-MB to genetic and pharmacological inhibition of HDAC1 and HDAC2.

Similarly to what observed in other solid tumors, such as colon, breast and lung^27^ carcinomas, HDAC1/2 levels are elevated in SHH-MB and their inhibition induces growth arrest and apoptosis, demonstrating the essential role of these proteins in tumor cell proliferation and viability. Different mechanisms have been proposed to explain the pro-tumorigenic role of these two deacetylases, most notably the hypo-acetylation and consequent transcriptional repression of the genes encoding the tumor suppressor p21 or DNA damage repair enzymes such as BRCA1 and ATR. Alternatively, direct deacetylation and inactivation of p53 or activating deacetylation of SP1 and C/EBPα with upregulation of oncogenes, such as Bcl-2^27^.

In this work we show that in SHH-MB cells, a key target underlying the HDAC1/2 pro-tumorigenic role is the Hh/Gli1 signaling itself. Indeed, Hh transcriptional output is significantly inhibited by inactivation of these two deacetylases, thereby leading to inhibition of cell proliferation and induction of cell death. Analysis of the three known HDACi-regulated targets (p53, Bcl-2 and p21) has revealed that, while the levels of p53 and Bcl-2 are unchanged, p21 is upregulated in mocetinostat-treated SHH MB cells. Since p21 is a key regulator of G1/S transition, this observation is in keeping with the cell cycle analysis, which shows an accumulation of cells in the G1 phase and consequent depletion in S phase. A similar p21 upregulation is observed upon treatment with Hh inhibitors, supporting the conclusion that mocetinostat and Hh inhibitors share common antitumor mechanisms. Importantly, in Gli1-deficient SHH MB cells, where p21 is upregulated and tumor cell growth is inhibited compared to controls, mocetinostat is unable to cause any further increase of p21 or inhibition of cell growth. This latter observation indicates that in these cells the mocetinostat-induced upregulation of p21 and inhibition of cell growth are Gli1-dependent.

Therefore, these data support the conclusion that the anti-cancer effect of mocetinostat achieved in Hh-addicted tumors can be attributed, at least in part, to the Gli1-dependent upregulation of p21. This mechanism appears to be specific for these cells, where Gli1 is a key tumorigenic driver, while in other tumor cells it is mediated by the alteration of different oncogenic effectors/signaling, to which each tumor is addicted.

Mechanistically, we show that the antitumor effect of HDAC1/2 inhibition is linked to their ability to induce Gli acetylation, a modification that limits the activity of these transcription factors, by preventing their promoter occupancy^16^.

In the cell model used in this study, the deletion of the upstream receptor Ptch1 causes hyperactivation of the signaling. Consequently, Gli1 is overexpressed and acts as a major tumorigenic driver, whereas Gli2 is barely detectable in these cells. Indeed, Gli1 ablation reduces the proliferation of these tumor cells, and abrogates the response to MGCD0103. The effect of this drug is tightly dependent on K518 acetylation, as demonstrated in MB cells stably expressing the non-acetylatabe Gli1 K518R mutant, where the proliferation rate does not change in response to MGCD0103.

As we show here, when administered to mouse models of SHH-MB, this drug crosses the blood-brain barrier, represses Hh signaling, inhibits cell proliferation and promotes apoptosis of tumor but not of normal cells. Importantly, the average survival of MB-prone mice is significantly increased by mocetinostat. Therefore, mocetinostat appears to be eligible as a new potential weapon against this class of malignancy, where effective pharmacological approaches are still missing. This evidence appears relevant since mocetinostat is a well-tolerated drug with marginal side effects. Indeed, it has been evaluated in phase II clinical trials for Hodgkin’s lymphoma with positive outcomes and a substantial improved response compared to the pan-HDAC inhibitor vorinostat^24^. The positive response obtained in this study encourages further work to inform us whether mocetinostat may be used in SHH-MB patients and enter clinical trials.

In a previous report it was observed that, in addition to HDAC1/2, HDAC6 is also upregulated in SHH-MB and its targeting leads to a substantial anti-tumor effect in mouse models of SHH-MB^29^. Interestingly, HDAC6 seems to exert its function independently of Gli acetylation, by acting at both receptor and downstream level, most likely by affecting Gli2/3 stability. Thus, it will be interesting to study if selective inhibition of HDAC1/2, combined to HDAC6, will result in additive anticancer effect on these tumors.

In summary, in this study we have demonstrated the ability of a clinically tested drug to counteract the growth of SHH-MB in mice, by acting as a downstream Hh inhibitor and inducing the acetylation of Gli1. Further studies in preclinical models and in clinical settings, alone and in combination with other drugs, are now required to determine whether this drug is eligible to enter into therapeutic protocols for the treatment of SHH-MB patients.

## Methods

### Cell culture, luciferase assays, transfection

Med1-MB cells, generated from a spontaneous tumor arising in a Ptch1^+/−^; lacZ mouse^18^,^19^ were kindly provided by Yoon-Jae Cho^18^ and were cultured in DMEM supplemented with 10% FBS, 1% Penicillin-Streptomycin, 1% Glutamine. Shh-Light II cells, WT, Ptch1^−/−^, Sufu^−/−^ MEF cells and GCPs were cultured as previously described^15^,^32^. Gli1/2^−/−^ MEFs were kindly provided by Matthias Lauth (Institute of Molecular Biology and Tumor Research (IMT), Marburg, Germany) and were cultured in DMEM supplemented with 10% FBS, 1% Penicillin-Streptomycin, 1% Glutamine. Primary medulloblastoma cell lines were obtained and cultured as previously described^33^. DAOY cells were purchased from the American Type Culture Collection (ATCC, Manassas, VA, USA) and cultured in MEM with Earl’s salts supplemented with 10% FBS, 2% Penicillin-Streptomycin, 1% Glutamine, 1% Non Essential Aminoacids, 1% Sodium Pyruvate. Luciferase assays on Shh-Light II cells and transfection in DAOY cells were performed as described^15^,^34^.

### Drugs

SAG was purchased from Adipogen, Life Sciences (Liestal, Switzerland). For Hh pathway activation, MEF cells were incubated overnight in serum-free medium, containing 1% BSA, and then treated with 200 nM SAG for the indicated time points. Kaad-cyclopamine was purchased from Millipore (cat. #239804), ATO and vorinostat were purchased from SIGMA (cat. #1010 and cat. #SML0061 respectively), mocetinostat (MGCD0103) was synthetized in house as a dihydrobromide salt as described^35^.

### RNA interference and Quantitative PCR

siRNA transfections of Med1-MB cells were performed with Lipofectamine 2000 (Invitrogen) for 6 hours. Cells were seeded on day 1, transfected twice (on day 2 and day 3) and then incubated for further 72 hours and re-seeded for treatments with MGCD0103 for further 48 h. The efficacy of knockdown was verified by Western Blot analysis at the end of the experiments. siRNAs were purchased from Dharmacon (Scrambled # D 001206-13-20 and mouse siGli1 # M-047917-01). Quantitative real time PCR was performed as described^15^ with the primers indicated for the different experiments (see Supplementary Table 1). Results were normalized by the levels of the housekeeping gene L32 and are represented as fold change, relative to control.

### Lentiviral-mediated shRNA knockdown

Lentiviral-mediated shRNA knockdown was performed as described previously^32^ using the MISSION shRNA methodology (Sigma). Virus titers were determined using the HIV-1 p24 ELISA (NEK050B001KT, Perkin-Elmer), according to the manufacturer’s instructions. The following pLKO.1 lentiviral vectors were used: TRCN0000039402 (shHDAC1), TRCN0000039397 (shHDAC2), shc002 (scrambled shRNA). Med1-MB cells and primary medulloblastoma cells were transduced with lentiviruses (MOI = 5), and incubated for the indicated times. Infected cells were selected with 5 μg/ml puromycin, where indicated.

### Western blotting

Cells were lysed using denaturing SDS/UREA buffer (50 mM Tris, 2% SDS, 10% Glycerol, 10 mM Na_4_P_2_O_7_, 100 mM NaF, 6 M Urea, 10 mM EDTA). Protein extracts were quantified, analyzed through SDS-PAGE and blotted onto a nitrocellulose membrane (Perkin Elmer). Membranes were blocked with 5% milk in Tris buffered saline with 0.1% Tween20, and incubated with the indicated antibodies.

### Cell proliferation and BrdU incorporation assays

Cell proliferation assay were performed in Med1-MB cells stably expressing shRNAs of HDAC1/2 or shSCR and cultured in DMEM supplemented with 5 μg/mL puromycin.

DAOY cells were transfected with plasmids encoding Gli1 WT or K518R mutant^15^. Stably transfected cells were selected with 0.8 mg/ml G418, seeded and treated with either 0.5 μM MGCD0103 or DMSO control for the indicated times. In each experiment cells were seeded in triplicates (2 × 10^4^ cells/well in multi-well 12) and counted every day for the indicated times. Incorporation of BrdU in Med1-MB or primary medulloblastoma cells, was performed by using the BrdU-labelling assay Kit (Roche) as previously described^15^,^32^. Each experiment was performed at least three times.

### Flow cytometry

Cell cycle profile was performed in Med1-MB cells treated with 0.5 μM MGCD0103 for 24 h, harvested, washed and fixed with 70% ethanol at +4 °C for 2 hours. Cells were then washed, treated with RNase A (40 μg/mL) and stained with propidium iodide (50 μg/mL). DNA content was measured by using a fluorescence-activated cell cytometer (Flow Cytometer C6, BD Accuri) and analyzed using BD Accuri C6 software. The Annexin V-propidium iodide double staining was performed in Med1-MB cells treated with 0.5 μM MGCD0103 for 48 h, and by using the Annexin V Apoptosis Detection Kit FITC (# 88-8005 e-Bioscience), according to the manufacturer’s instructions. Experiments were analyzed as described above.

### Immunohistochemistry and Tunel assay

Tissue samples were fixed and paraffinized as described^32^,^36^. Sections from paraffin-embedded tissues were deparaffinized with xylol, fixed with decreasing ethanol concentrations, blocked and incubated with the indicated antibodies: anti Gli1 1:100, anti acetyl-Gli1-Lys518 1:50, anti Ki67 1:100.

Sections were counterstained with hematoxylin to check the quality of slides.

Apoptotic cell death on paraffin embebbed sections was evaluated by Tunel assay (Tdt-mediated dUTP-nick end labeling) by using in situ cell death detection kit, POD (Roche # 11684817910), accordingly to the manufacturer’s instructions, and counterstained with hematoxylin.

### Animal studies

Tumor allografts were performed as described^32^,^33^. Briefly, athymic CD1 nude mice were injected on each flank with 2 × 10^6^ primary SHH-MB cells, obtained from tumors originated in Math1-Cre/Ptc^fl/fl^ mice, or with 2 × 10^6^ Med1-MB cells stably expressing shRNA-SCR or shRNA-HDAC1/2. In pharmacological experiments, when the tumor reached the volume of 100 mm^3^, mice were treated with 170 mg/kg MGCD0103^23^ *per os.* Tumors were measured using calipers, with the following formula: V = (L × W^2^)/2.

For acute treatments, symptomatic Math1-Cre/Ptc^fl/fl^ mice or WT littermates were injected subcutaneously with 170 mg/kg MGCD0103 or control DMSO for 6 hours and then western blot or RT-QPCR analyses were performed.

Animal studies were performed according to the European Community Council Directive 2010/63/EU and were approved by the local Ethical Committee for Animal Experiments of the Sapienza University of Rome.

### Antibodies

The following antibodies and dilution were used: Gli1 (# 2643 S Cell Signaling) 1:1000, Gli1 H300 Santa Cruz Biotechnology (sc-20687) 1:100, Gli2 (# AF3635 R&D) 1:1000, HDAC1 (ab31263-100 AbCam) 1:2000, HDAC2 (sc-7899X Santa Cruz) 1:2000, Vinculin (sc-7649 Santa Cruz) 1:1000, Actin (sc-1616 Santa Cruz) 1:1000, PARP (# 9542 S Cell Signaling) 1:1000, Tubulin (Santa Cruz Biotechnology sc-8035) 1:1000, Cleaved Caspase 3 (# 9661 S Cell Signaling) 1:1000, p21 (sc-817 Santa Cruz) 1:1000, p53 (#2524 Cell signaling) 1:1000, Bcl-2 (sc-492 Santa Cruz) 1:1000, Acetyl Histone H3 (# 06-599 Millipore) 1:1000, Ki67 (MA5-14520 Pierce) 1:100, Flag-M2 Peroxidase (A8592 Sigma) 1:5000, AcGli1 was described previously ref. 16 and was used at 1:50 dilution for IHC and 1:500 for western blot.

### Statistical analysis

All experiments were performed at least three times and all results represented are expressed as the average +/− SD. Statistical analysis was performed using StatView 4.1 software (Abacus Concepts, Berkeley, CA). Statistical differences between the means were analyzed by the Mann-Whitney *U* test for non-parametric values. Differences were considered significant at *p < 0.05.

## Additional Information

**How to cite this article:** Coni, S. *et al*. Selective targeting of HDAC1/2 elicits anticancer effects through Gli1 acetylation in preclinical models of SHH Medulloblastoma. *Sci. Rep.* **7**, 44079; doi: 10.1038/srep44079 (2017).

**Publisher's note:** Springer Nature remains neutral with regard to jurisdictional claims in published maps and institutional affiliations.

## Supplementary Material

Supplementary Information
