# Corrigendum: Selective targeting of HDAC1/2 elicits anticancer effects through Gli1 acetylation in preclinical models of SHH Medulloblastoma

**DOI:** 10.1038/srep46645

**Published:** 2017-04-21

**Authors:** Sonia Coni, Anna Barbara Mancuso, Laura Di Magno, Giulia Sdruscia, Simona Manni, Silvia Maria Serrao, Dante Rotili, Eleonora Spiombi, Francesca Bufalieri, Marialaura Petroni, Monika Kusio-Kobialka, Enrico De Smaele, Elisabetta Ferretti, Carlo Capalbo, Antonello Mai, Pawel Niewiadomski, Isabella Screpanti, Lucia Di Marcotullio, Gianluca Canettieri

Scientific Reports 7: Article number: 44079; 10.1038/srep44079 published online: 03 09 2017; updated: 04 21 2017.

In this Article, Monika Kusio-Kobialka and Pawel Niewiadomski are incorrectly listed as being affiliated with ‘Department of Cell Biology, Nencki Institute of Experimental Biology, 02-093, Warszawa, Poland’. The correct affiliation is listed below:

Centre of New Technologies, University of Warsaw, 02-097 Warszawa, Poland.

