# Supplementary material for: Selective targeting of HDAC1/2 elicits anticancer effects through Gli1 acetylation in preclinical models of SHH Medulloblastoma
Source: Sci Rep. 2017 Mar 9;7:44079. doi: 10.1038/srep44079 (PMC5343431; doi:10.1038/srep44079)
Supplement: Supplementary Information [file srep44079-s1.pdf]

Selective targeting of HDAC1/2 elicits anticancer effects through Gli1 acetylation in preclinical models of SHH Medulloblastoma

Sonia Coni, Anna Barbara Mancuso, Laura Di Magno, Giulia Sdruscia, Simona Manni, Silvia Maria Serrao, Dante Rotili, Eleonora Spiombi, Francesca Bufalieri, Marialaura Petroni, Monika Kusio-Kobialka, Enrico De Smaele, Elisabetta Ferretti, Carlo Capalbo, Antonello Mai, Pawel Niewiadomski, Isabella Screpanti, Lucia Di Marcotullio and Gianluca Canettieri

Supplementary figures, tables and figure legends

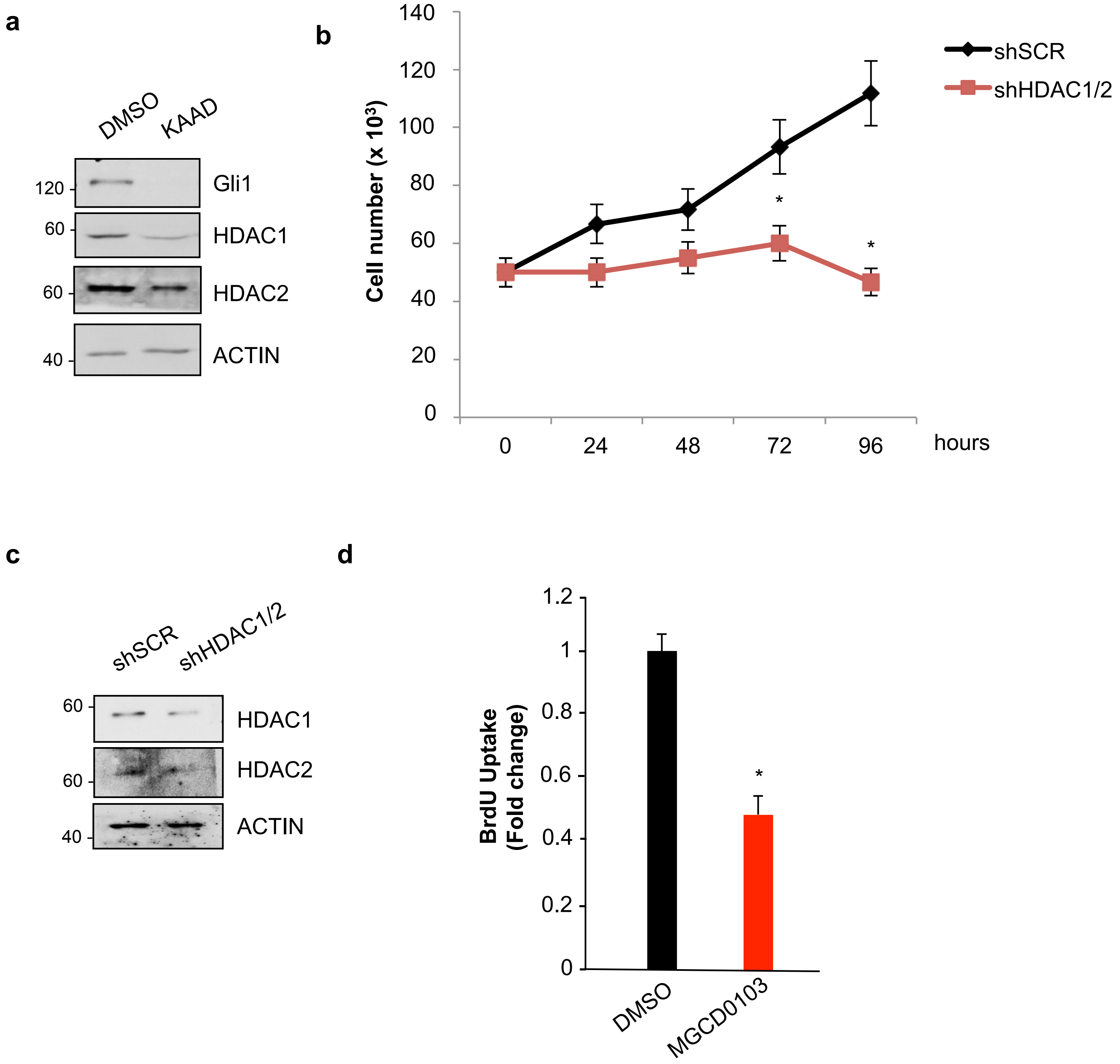

Supplementary Figure S1

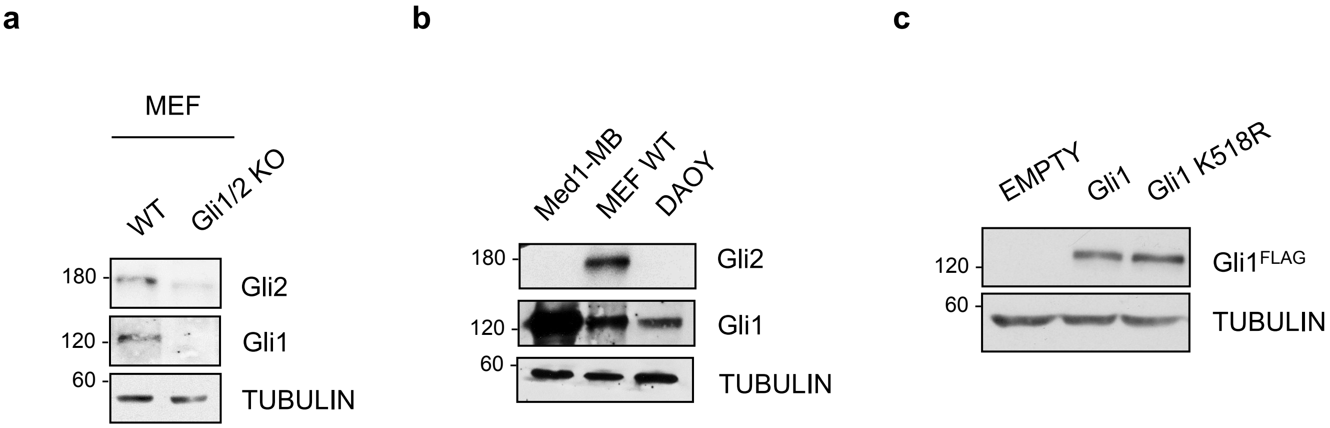

Supplementary Figure S2

a

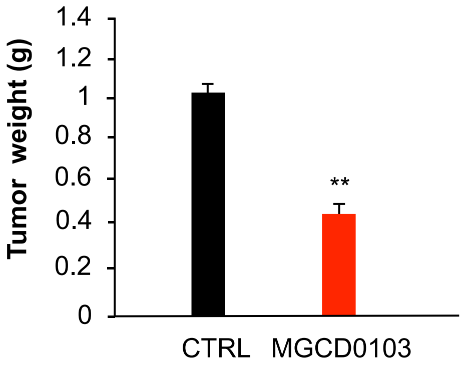

b

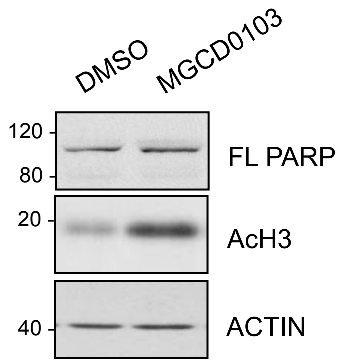

Supplementary Figure S3

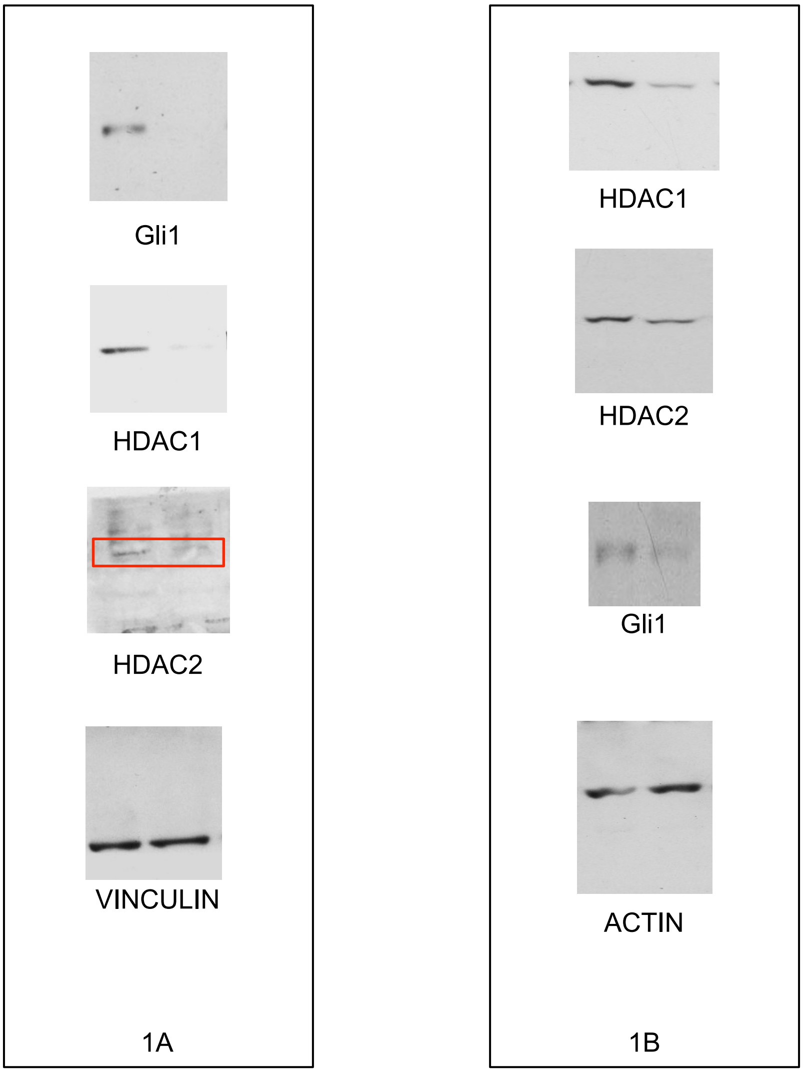

Supplementary Figure S4

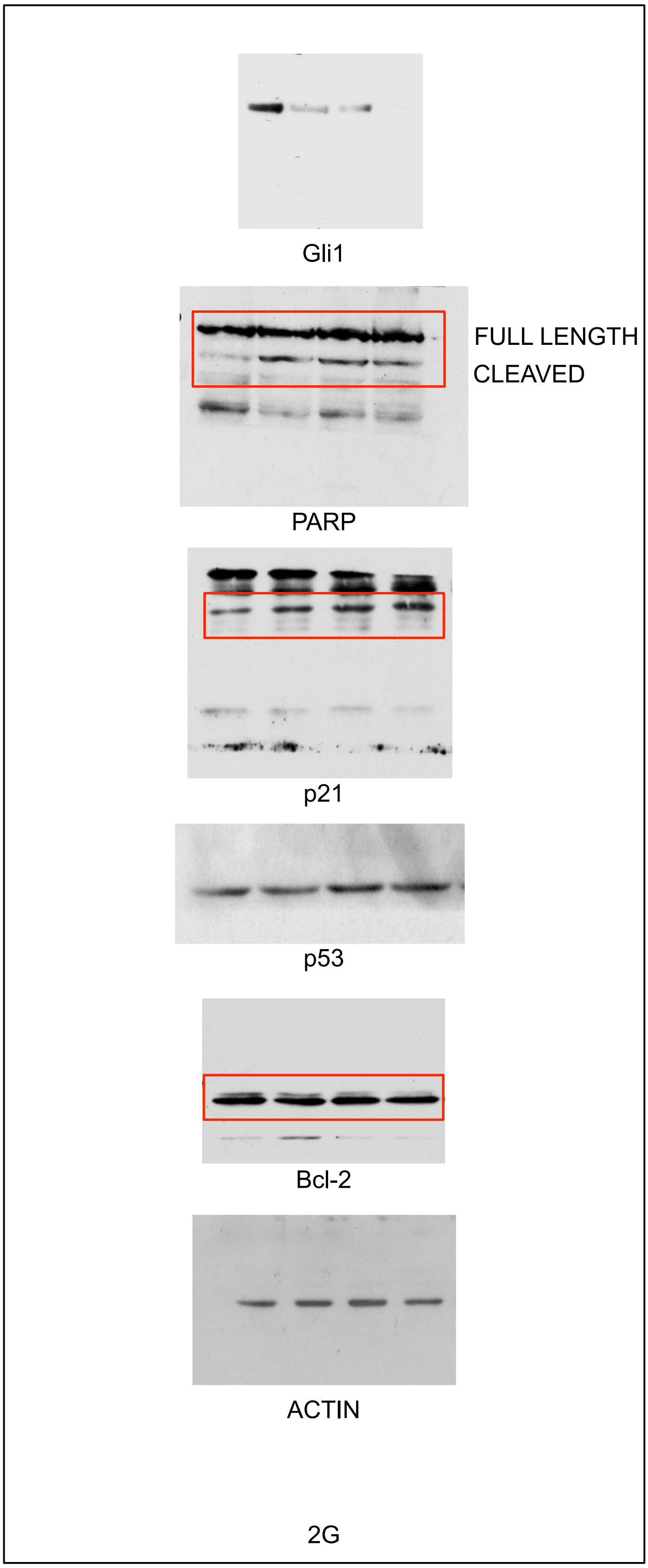

Supplementary Figure S5

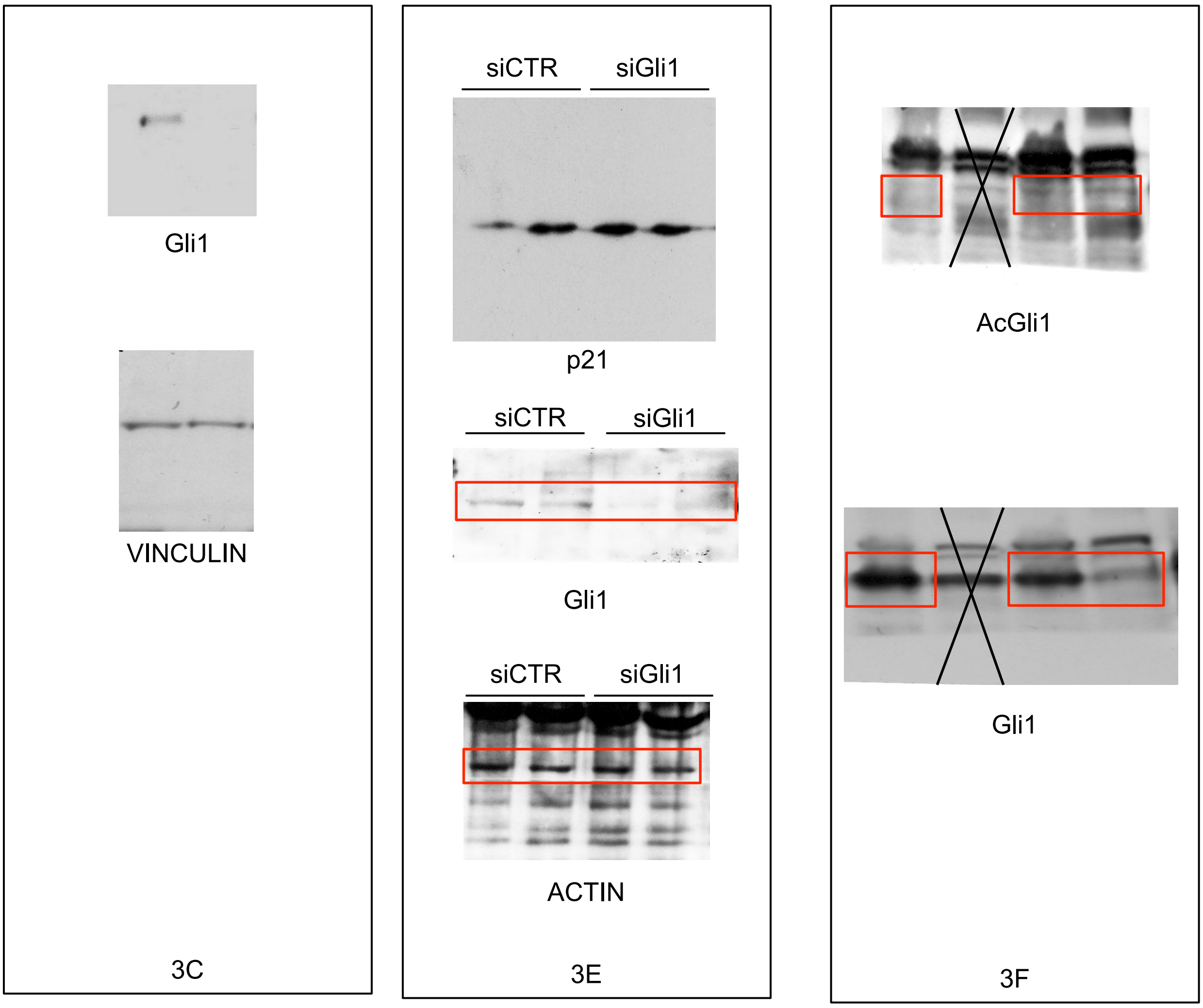

Supplementary Figure S6

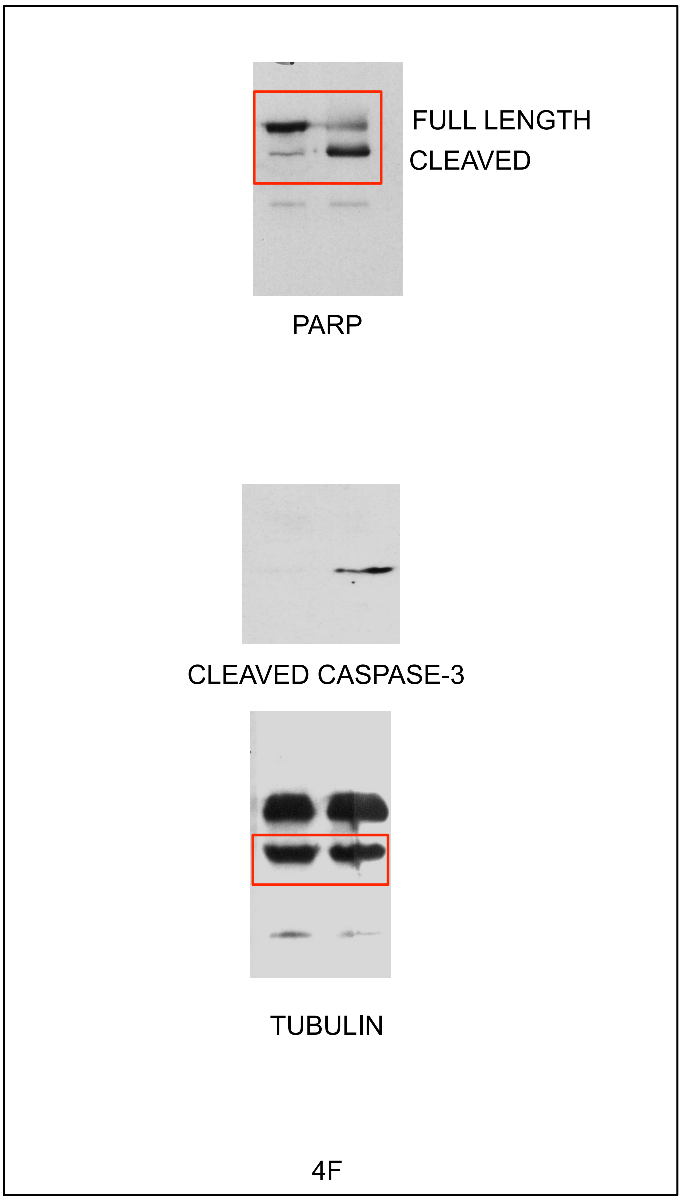

Supplementary Figure S7

**Sybr Green oligonucleotides**	**Sequence**
**mPtch1**	**FW: GCATTGGCAGGAGGAGTTGA**<br>**RW: AGTCATTAACTGGAACATGGTTTGC**
**mPtch2**	**FW: CAACATGGTCGCCTTTTTCA**<br>**RW: ACCACTATGGCTGCCTGCA**
**mCyclinD1**	**FW: TCCGCAAGCATGCACAGA**<br>**RW: AGGGTGGGTTGGAAATGAACT**
**mCyclinD2**	**FW: TTTCAAGTGCGTGCAGAAGG**<br>**RW: CCAGCCAAGAAACGGTCCAG**
**mGli1**	**FW: AAGCCAACTTTATGTCAGGG**<br>**RW: AGAGCCCGCTTCTTTCTTAA**
**mL32**	**FW: AGAGGTGCTGGGAGCTGCTA**<br>**RW: GATGGATGGTCTCTGGACGG**
**mHDAC1**	**FW: TCGCTGCTGGACTTACGAAA**<br>**RW: TGTAGGGCAGCTCATTAGGGA**
**mHDAC2**	**FW: TCCGGTGTTTGATGGACTCTT**<br>**RW: TCACAGCCCCAGCAACTGA**
**mN-Myc**	**FW: AAGCGTGGATTATGACAGGAAGTC**<br>**RW: CTTGTCAACAACGCCAGTTTCCAC**

Supplementary Table 1

Figure S1: HDAC1 and HDAC2 inhibition impairs Mb primary tumors growth *in vitro*. (**a**) Western Blot analysis of Gli1, HDAC1, HDAC2 protein levels in primary MB cells treated with 1μM Kaad-cyclopamine or DMSO for 72 hours. Actin, loading control. (**b**) Growth curve of primary SHH MB cells stably transduced with lentiviruses expressing HDAC1/2 or scrambled shRNAs. Experiments were performed in triplicate for the indicated times. (**c**) Western blot analysis to verify knockdown efficacy. (**d**) BrdU incorporation assay in primary MB cells treated for 48 hours with 0.5 μM MGCD0103 or DMSO. *p<0,05.

Figure S2: Gli levels in cell lines used. (**a**) Gli1 and Gli2 protein levels on MEF cells WT or Gli1/ Gli2 KO. Tubulin, loading control. (**b**) Western Blot analysis of Gli1 and Gli2 protein levels in Med1-MB, MEF and DAOY cells. Tubulin, loading control. (**c**) Western blot analysis in DAOY cells stably expressing Flag-tagged Gli1 WT and K518R (figure 3h). Tubulin, loading control.

Figure S3: Mocetinostat effect in mouse models of SHH MB. (**a**) Average tumor weight of tumor masses explanted at the end of the allograft experiment shown in figure 4a. (**b**) Western blot analysis on cerebella from WT littermates from figure 4f. Mice were subcutaneusly injected with DMSO or 170mg/Kg MGCD0103 for 6 hours (n= 3 CTRL; n=3 MGCD0103). PARP, AcH3 and actin levels are shown.

Figure S4: Uncropped Western blots related to main Fig.1.

Figure S5: Uncropped Western blots related to main Fig.2.

Figure S6: Uncropped Western blots related to main Fig.3.

Figure S7: Uncropped Western blots related to main Fig.4.

Supplementary table 1. Sybr Green oligonucleotides designed and used in the experiments.
